# Penicillosis with lymphadenopathy mimicing tuberculosis

**DOI:** 10.1186/1471-2334-14-S3-P30

**Published:** 2014-05-27

**Authors:** Biswadeep Borthakur, Lahari Saikia, Dhrubajyoti Borah, Sreemanta Madhab Barua, Ratan Kumar Kotokey, BB Rewari, Partha patim Das, Divya Singh

**Affiliations:** 1 Assam Medical College, Dibrugarh, Assam, India; 2 National AIDS Control Organization, India

## Background

In India, although the maximum cases of penicillosis caused by *Penicillium marneffei* have been reported from Manipur, cases in the bordering states like Assam and Nagaland are not very rare. From past 2 years we have been receiving significant number of cases at Assam.

## Methods

We describe two similar cases of disseminated penicillosis presenting with fever and weight loss with multiple mesenteric lymphadenopathy and skin lesion, clinically mimicking extrapulmonary tuberculosis. Penicilliosis was diagnosed by usg guided FNAC of mesenteric LN. *P. marneffei* was confirmed by microscopy and cultured, onto sabouraud dextrose agar. The cases were treated with oral itraconazole along followed by HAART two weeks later. However the lymph nodes enlarged (IRIS), so we withheld HAART for 2 weeks continuing itraconazole. After one and half months of treatment the skin lesions healed and mesenteric Lymph-Nodes disappeared completely with significant weight gain.

## Conclusion

Many cases of disseminated penicilliosis have been misdiagnosed as tuberculosis, where the fungal disease is prevalent. Both infections have similar symptomology. Perhaps these cases of disseminated penicilliosis with IRIS which responded only with Itraconazole without using Amphotericin-B. Itraconazole can be a safer option than amphotericin-B HIV with lymphadenopathy, penicilliosis has to be brought into consideration in endemic area.

